# Sex and Health Disparities Impacts on Survival Rates for Patients With Major Salivary Gland Tumors

**DOI:** 10.1002/cam4.71510

**Published:** 2026-02-05

**Authors:** Andrew R. Cunningham, Obaid U. Khurram, Hailey C. Lewis, Nathan Barefoot, Andrew W. Ju, Sean P. Holmes, M. Sean Peach

**Affiliations:** ^1^ Brody School of Medicine at East Carolina University Greenville North Carolina USA; ^2^ Department of Physiology and Biomedical Engineering Mayo Clinic Rochester Minnesota USA; ^3^ Department of Radiation Oncology, Brody School of Medicine East Carolina University Greenville North Carolina USA; ^4^ Eastern Carolina ENT‐ Head and Neck Surgery Greenville North Carolina USA

**Keywords:** cancer, cancer registry, health disparities, major salivary gland tumor, salivary gland malignancy, salivary neoplasm, SEER database

## Abstract

**Objective:**

To evaluate demographic and clinical factors influencing survival in patients with major salivary gland tumors (MSGTs) using the surveillance, epidemiology, and end results (SEER) database.

**Study Design:**

A retrospective cohort study using SEER data from 2000 to 2021.

**Setting:**

Data were collected from the SEER registry, a comprehensive database capturing cancer statistics across the United States.

**Methods:**

Patients diagnosed with major salivary gland malignancies were analyzed for demographic factors, including age, sex, race, marital status, and income, as well as clinical variables, including tumor grade and site. Stratification by sex was done to assess sex‐specific outcomes.

**Results:**

The cohort included 13,869 patients. Key findings include that older age and male sex were associated with worse survival outcomes. Single patients had better survival than married individuals, likely due to younger age at diagnosis. Additionally, higher income and Asian/Pacific Islander race conferred a survival advantage. There were disparate impacts between males and females in income, marital status, and race, with higher income and marriage providing a survival advantage for male patients but none for females. Additionally, Black females but not Black males, and widowed females but not widowed males, had slightly increased hazard ratios compared to White individuals.

**Conclusion:**

Men and women experience disparate effects that impact survival and outcomes in major salivary gland tumors. These findings highlight the need for targeted interventions to address disparities in care and improve survival outcomes while emphasizing the importance of further research to understand the underlying mechanisms driving these disparities.

## 1 Introduction

Salivary gland tumors account for 5% of all head and neck cancers and 0.5% of all malignancies [1]. Three of these salivary glands compose the major salivary glands, including the parotid, submandibular, and sublingual glands. Salivary gland malignancies present complexities morphologically, histologically, and biologically due to their uncommon and diverse nature [2]. Over 20 histological subtypes have been reported in adult populations, as recognized by the 5th edition of the World Health Organization head and neck tumor classifications [3]. Although classification is complicated by considerable morphological diversity [4], mucoepidermoid carcinoma, adenoid cystic carcinoma, and polymorphic adenocarcinoma constitute the majority of malignant cases [5, 6, 7].

While the influence of demographic factors on survival rates in head and neck carcinomas has been increasingly studied among these variants, studies have not sufficiently addressed the rising gap in outcomes between men and women. Previous analyses of the surveillance, epidemiology, and end results (SEER) database have generally characterized the population of salivary gland malignancies and noted the importance of variables such as age, income, sex, and marital status [8, 9, 10]. In the analysis of SEER data from 1973 to 2009, Olarte et al. showed that younger age, female sex, and marriage conferred a survival benefit for individuals diagnosed with major salivary gland tumors (MSGTs) [9]. In individuals diagnosed with parotid gland cancer, Stubbs et al. found that women, Asians, and a higher education level conferred a survival benefit in patients [10]. These studies and others have shown that females have consistently shown better survival than males, seen in both MSGTs and other head and neck cancers. Saraswathula et al. utilized the SEER database and found that patients with private insurance had better survival than those with Medicare and Medicaid [11]. Megwalu et al. also completed a SEER analysis from 2004 to 2012 on oropharyngeal squamous cell carcinoma and found that residing in a low socioeconomic status (SES) county was associated with worse overall and disease‐specific survival when controlling for race, age, sex, marital status, year of diagnosis, site, stage, grade, and treatment modality [8]. These studies suggest that lack of access to healthcare and lower SES confer decreased survival in head and neck cancers.

While previous studies have explored various demographic factors, multivariate analyses that account for sex‐specific differences in survival are lacking. To address this gap in the literature, we evaluated the impact of a major salivary gland tumor diagnosis on survival with respect to a variety of demographic factors stratified by sex. Our goal was to evaluate the impacts of demographics, including marital status, race, SES, and age, on males versus females to understand if additional factors contribute to the growing differences in outcomes between sexes. Previous studies suggest that females tend to seek medical care at earlier stages of disease progression, leading to earlier diagnoses and potentially less advanced tumor grades at diagnosis [12]. We hypothesized that females would have better overall survival than males, in part due to presenting with lower tumor grade at diagnosis.

## 2 Methods

### 2.1 Data Source and Patient Selection

A retrospective cohort study utilizing the SEER Program was conducted for patients with a diagnosis of major salivary gland malignancy. Specifically, the 2000–2021 Incidence—Research Plus Data, 17 Registries (April 2023 release) was queried via a case listing session in SEER*Stat [13]. Inclusion criteria was Site and Morphology Primary Site of C07.9—Parotid Gland, C08.0—Submandibular Gland, C08.1—Sublingual Gland, C080.8—Overlapping lesion of major salivary glands or C08.9—Major Salivary Gland, NOS and the International Classification of Diseases for Oncology, 3rd edition (ICD‐O‐3) Behavior Code equal to “Malignant.”

### 2.2 Variables/Outcome Data and Analysis

Demographics (age, sex, race, marital status, SES utilizing county‐level household income), disease grade, and survival from diagnosis were collected. Data were analyzed in Python Version 3.12. We used several Python libraries for data management, visualization, and statistical modeling. Data preprocessing was performed using *pandas* for DataFrame manipulations and *numpy* for numerical operations. Data visualization was conducted using *matplotlib* and *seaborn*. Statistical analyses included Kaplan–Meier (KM) survival analysis and Cox Proportional Hazards (PH) modeling, implemented via the *lifelines* package and detailed below. For all statistical assessments, a *p* value of < 0.05 was selected as the cutoff for statistical significance. 95% confidence intervals (CI) are reported in brackets. For analysis of SES, county‐level income bins were divided into approximate tertiles based on the numerical midpoint across each of the income bins reported in the SEER database.

### 2.3 KM Survival Curves

KM survival analysis was performed to estimate survival probabilities for various subgroups, including histological types, races, socioeconomic tertiles, sex, marital statuses, and primary tumor sites. The KM curves were generated using the *KaplanMeierFitter* from *lifelines*. Survival times (in months) were right‐censored based on the patient's vital status. A 95% confidence interval was also determined and graphed alongside the survival curves.

### 2.4 Assessment of PH Assumption

To ensure the validity of the Cox model, we tested whether the PH assumption was met, as this is fundamental to the reliable interpretation of hazard ratios over time. This assumption was evaluated using Schoenfeld residuals, which were assessed for each covariate to identify deviations. We applied a global test and individual tests for each variable, with *p* values < 0.05 indicating potential violations. Sex, tumor grade, site, and histology, all of which are reported in Table 1, did not meet the PH assumption. To account for these violations, data were stratified by sex. Stratification by sex led to only tumor grade level high (*p* < 0.001), grade level unknown (*p* = 0.038), and primary tumor site major salivary gland (not otherwise specified—NOS; *p* = 0.018), still violating the PH assumption. Given the importance of tumor grade, site, and histology in clinical assessments, these variables were still included in the Cox regression models despite the violation of the PH assumption.

**TABLE 1:** Demographics and clinical characteristics of patients with primary major salivary carcinomas.

Variable	Total (*n* = 13,869, %)
Age	
< 65 years	8275 (59.67%)
≥ 65 years	5594 (40.33%)
Sex	
Male	6669 (48.09%)
Female	7200 (51.91%)
Race	
White	10,735 (77.40%)
Black	1479 (10.66%)
Asian/Pacific Islander	1401 (10.10%)
American Indian/Alaska Native	100 (0.72%)
Other or unknown	154 (1.11%)
Marital status	
Married	7718 (55.65%)
Single (never married)	2798 (20.17%)
Widowed	1255 (9.05%)
Separated/divorced	1211 (8.73%)
Domestic partner	36 (0.26%)
Unknown	851 (6.14%)
Household income	
Lower tertile (< $72,500)	5999 (43.26%)
Middle tertile (≥ $72,500 and < $87,500)	3511 (25.32%)
Upper tertile (≥ $87,500)	4359 (31.43%)
Grade level	
Low	1858 (13.40%)
Intermediate	2920 (21.05%)
High	2528 (18.23%)
Unknown	6563 (47.32%)
Primary site	
Parotid gland	10,963 (79.05%)
Submandibular gland	1892 (13.64%)
Sublingual gland	209 (1.51%)
Overlapping lesion of the major salivary glands	11 (0.079%)
Major salivary gland, NOS	794 (5.73%)
Histology	
Adenocarcinoma	5129 (36.98%)
Mucoepidermoid carcinoma	5104 (36.80%)
Adenoid cystic carcinoma	2252 (16.24%)
Other rare types	1306 (9.42%)
Mixed subtypes	78 (0.56%)

## 3 Results

### 3.1 Cohort Characteristics

**FIGURE 1:**
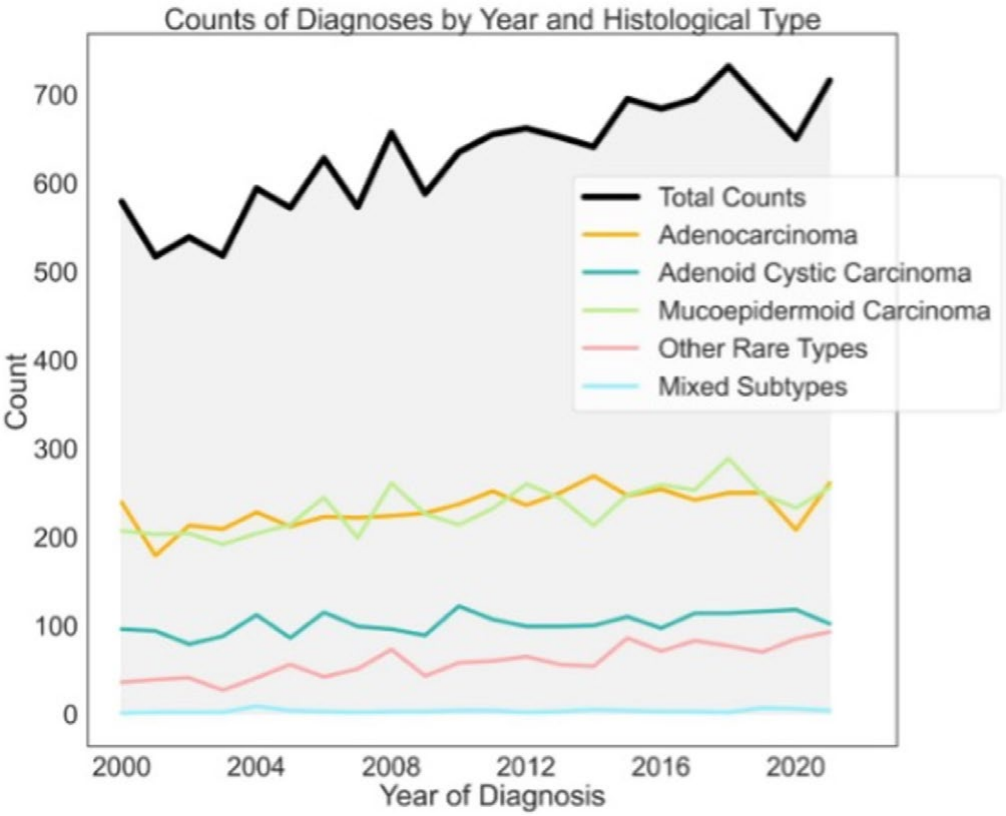
Incidence of major salivary gland tumors over the previous 20 years.

We identified 13,869 patients with malignancy of the major salivary glands from the available dataset. There continues to be an increase in the incidence of major salivary gland cancers year over year (Figure 1). Adenocarcinoma accounted for 39.98% of cases, closely followed by MEC at 36.80% and then ACC at 16.25%. Other types comprised less than 10% of cases collectively.

**FIGURE 2:**
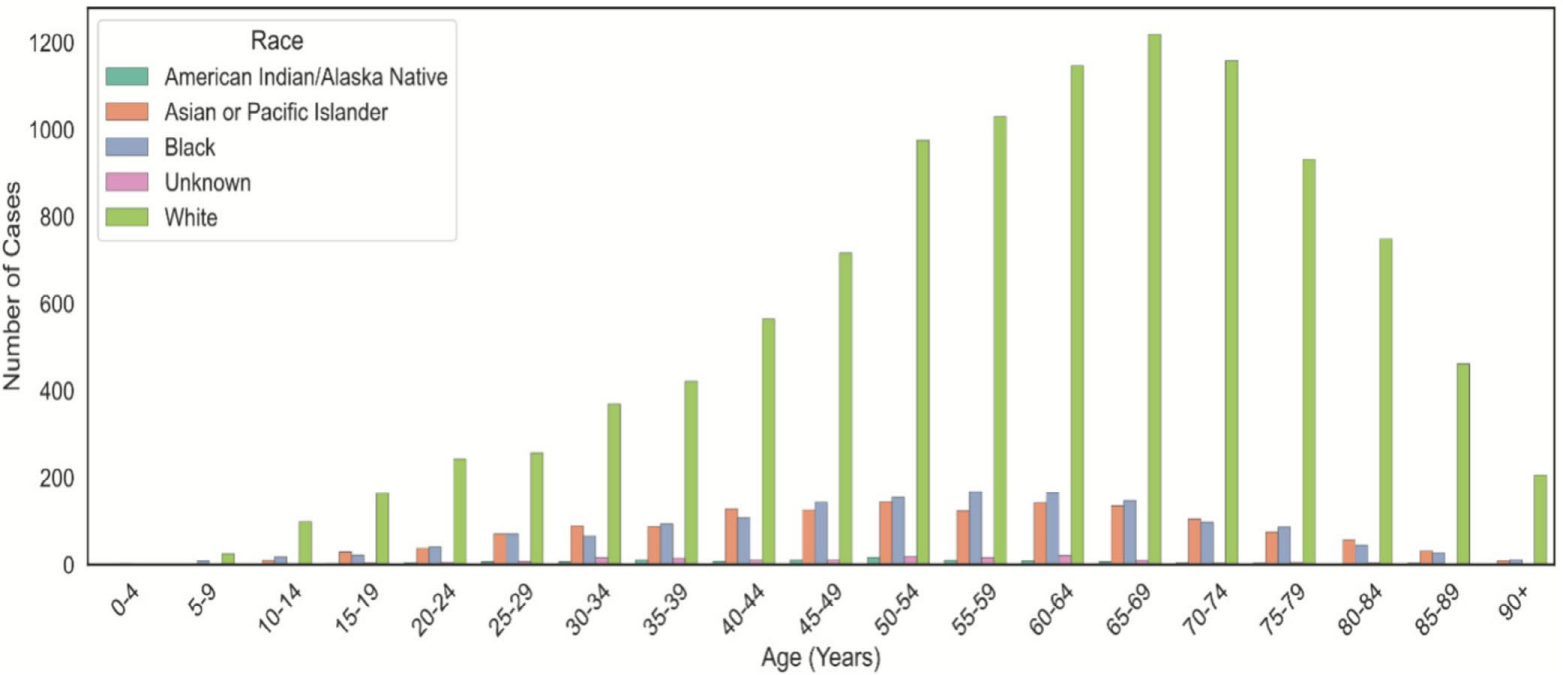
Graphical representation of the demographic variables, including distribution of race as a function of age at time of initial diagnosis.

Ten‐year overall survival was 62.45% [Range: 61.29, 63.60] for the entire cohort. Median age in years of the cohort was 60 years. Across all age groups, 77.40% of cases reported in the SEER registry comprised MSGTs in White individuals (Figure 2). Data were slightly imbalanced between sexes, with females comprising 51.91% of cases. More than 79.05% of the cases originated from the parotid gland, followed by the submandibular gland at 13.64%. The SEER pathological stage classification of most cases was listed as unknown at 47.32% of cases. Table 1 demonstrates a breakdown of the cohort by the analyzed demographic and characteristic variables.

### 3.2 Survival Analysis

**FIGURE 3:**
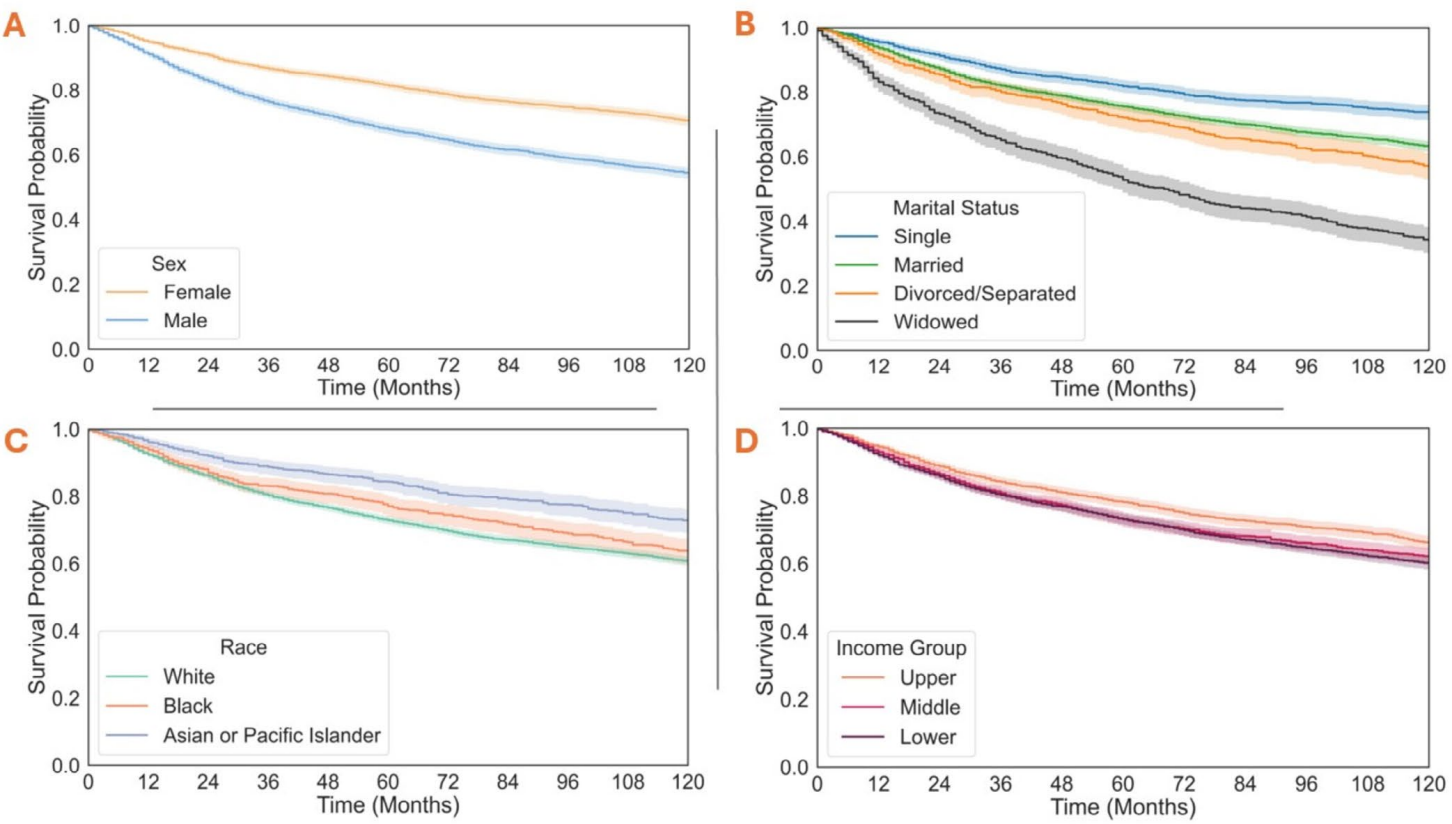
Kaplan–Meir Plots of the 10‐year survival across demographic variables. Solid lines demonstrate the KM estimates with shaded areas representing 95% confidence intervals.

Sex, race, marital status, and county‐level income were analyzed as seen in Figure 3. Female sex (Figure 3A) is associated with improved survival. Relationship status (Figure 3B) of single shows a small benefit to survival over married, with a significant decline in survival for widowed individuals. Analyzing race (Figure 3C), Asian/Pacific Islanders had the best survival, while White individuals had the worst. In household income (Figure 3D), only the top tertile provided a small benefit to survival. Survival associated with site, grade, and histology is provided in Figure S1. Numbers at risk for both Figures 3 and SS1 are provided in Table S1.

### 3.3 Multivariate Analysis

**FIGURE 4:**
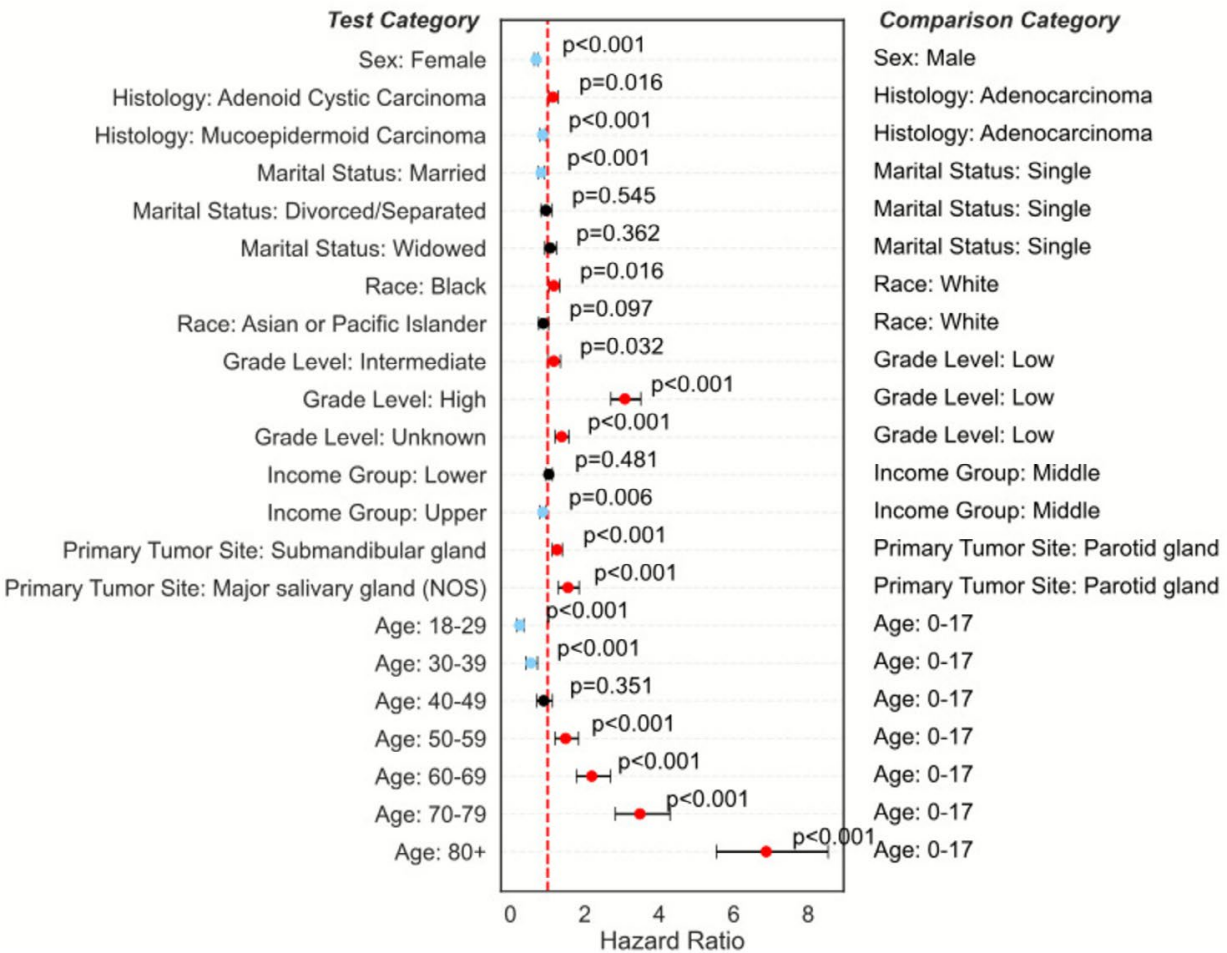
Cox Proportional Hazards (PH) model plotted on a forest plot to demonstrate the impacts of various demographic factors on survival. Blue indicates factors with significant survival benefit, while red dots indicate significant survival detriment.

Multivariate analysis using an adjusted Cox PH model was completed across various demographics to determine risk factors that impact survival for patients (Figure 4). Female sex, being married, being of Asian/Pacific Islander race, having a higher income, and age less than 40 years were associated with reduced risk for developing salivary gland malignancy. Age greater than 50 was associated with increased risk of malignancy.

Further analysis was also done to compare race and income, showing a stark difference between a patient's race and income in the cohort (Table 2). 58.1% of Asian/Pacific Islander patients were above the median income group, while 44.1% of white, 55.0% of black, and 47% of American Indian/Alaska Natives were below the median income group. A heatmap demonstrating the correlation among all variables is available in Figure S2.

**TABLE 2:** Income stratification by race: Proportion of salivary gland tumor patients below, at, and above the median income.

Race	Lower	Middle	Upper
American Indian/Alaska Native	47 (47.0%)	28 (28.0%)	25 (25.0%)
Asian or Pacific Islander	342 (24.4%)	245 (17.5%)	814 (58.1%)
Black	814 (55.0%)	375 (25.4%)	290 (19.6%)
Unknown	60 (39.0%)	41 (26.6%)	53 (34.4%)
White	4736 (44.1%)	2822 (26.3%)	3177 (29.6%)

### 3.4 Male Versus Female Stratification

**FIGURE 5:**
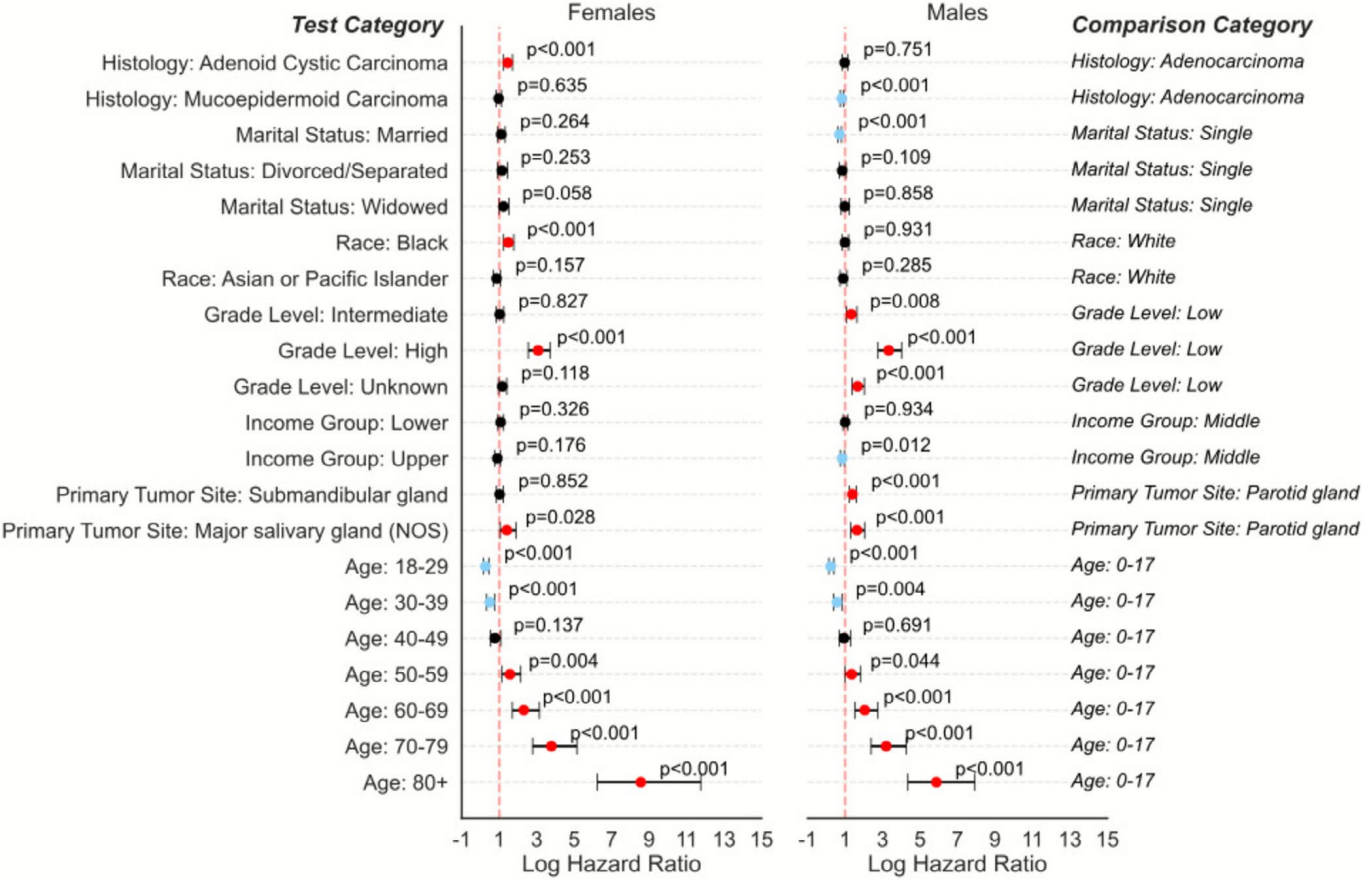
Cox Proportional Hazards (PH) model plotted on forest plot stratified by sex across marital status, race, income, and age at diagnosis. Blue indicates factors with significant survival benefit, while red dots indicate significant survival detriment.

Given the violation of sex in the PH assumptions, we reassessed the covariates stratified by sex to determine the effects, if any, of sex on salivary gland malignancies. The Cox PH model was split by male versus female. Female sex was reassessed across marital status, race, income, and age in Figure 5.

Age < 40yo (excluding < 18yo) was a protective factor for both males and females. Compared to individuals who were single (never married), married males had a slightly lower risk of death, but married females did not. Females who were widowed and females who identified as Black had slightly increased hazard ratios. Increased income was protective for men but not for women.

**FIGURE 6:**
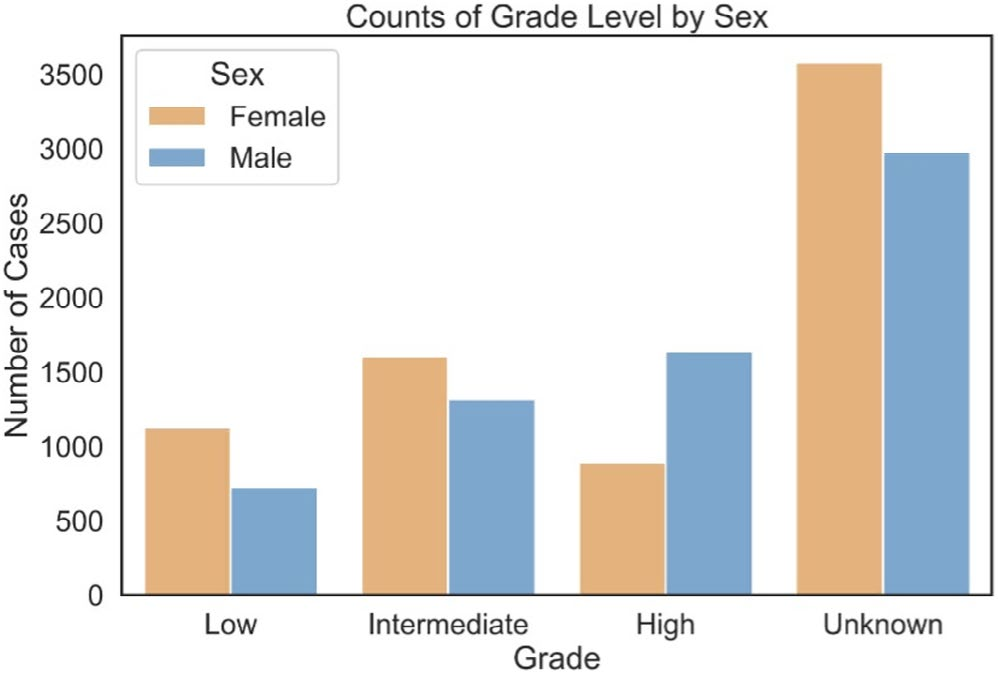
Histogram of the distribution of tumor stage stratified by sex.

Further analysis of the tumor characteristics in males and females demonstrated a differing distribution of tumor grade. Females tended to present with a higher proportion of lower‐stage disease than men, as seen in Figure 6.

## 4 Discussion

This updated analysis of MSGTs builds upon prior work by Olarte et al. [9] by not only expanding the dataset and follow‐up time but also addressing critical limitations in their approach. Our study incorporates nearly 3200 additional total patients over a 12‐year extended follow‐up period (2000–2021 vs. 1973–2009 in Olarte et al.) and leverages the detailed data on demographic and socioeconomic factors influencing survival in MSGTs. Over a decade's worth of additional data offers a critical update on survival trends, especially as MSGT incidence continues to rise (Figure 1). Given the documented rise in healthcare disparities and demographic complexity, this updated analysis is both timely and essential.

Olarte et al. primarily employed standard Cox PH modeling and KM analysis with broad demographic categories (sex, race as “White,” “Black,” or “Other,” and marital status limited to “married” and “single”). Our revised study not only increases sample size and follow‐up duration but also explicitly tests the Cox PH assumptions, identifying violations and stratifying the analysis accordingly—especially by sex. This nuanced approach enhances the validity and interpretability of the findings, ensuring that statistical assumptions underpinning hazard models are appropriately addressed.

While Olarte et al. noted that female sex conferred a survival advantage, they did not analyze interaction effects or stratify the survival models by sex. Our findings also initially demonstrate that females have superior survival to their male counterparts (Figure 4). This was also previously reported in the SEER database [9]. Further, work by Mimica et al. utilizing a cohort of 867 patients at their institutional database, found that women had superior survival compared to men [14]. Other large databases also demonstrated that female patients have better survival across most cancer sites [15]. Our study systematically stratified the Cox models to reveal sex‐specific patterns in how marital status, race, and income influence outcomes (Figure 5). These results may also be explained by women having a higher incidence of lower disease stage (Figure 6) and a lower incidence of nodal metastasis at the time of diagnosis. This observation was also noted in a study by Jung et al. analyzing the Korean National Registry Data, showing women had a higher incidence of Stage I tumors and a significantly higher proportion of localized head and neck cancers than male patients (42.3% vs. 28.1%) [15]. New staging variables in the SEER database may be able to further determine whether this finding is also true at the national level as it is entered, but this could not be analyzed because 47.32% of pathological stage data are currently missing.

Furthermore, we demonstrate that the relationship between marital status and survival is significantly impacted by the age of the groups. For example, widowed individuals had the worst survival but were also the oldest, while single individuals had better survival but were significantly younger at diagnosis. Increased age alone was associated with an increased likelihood of salivary gland tumor development. Previous studies utilizing the SEER database have also reported that overall and disease‐specific survival outcomes were lower with increasing age, concluding based on univariate and multivariate analysis that age was an independent predictor of survival outcome for those with major salivary gland malignancies [9, 16]. We further showed that, regardless of sex, those > 50 years old had worse outcomes and those < 40 years old had better outcomes (Figure 5). Age seems to have a significant impact on marital status analysis, where our primary analysis showed single individuals living longer than married individuals. As Table S2 demonstrates, the mean and median age of the single (never married) cohort is over 10 years younger than the married cohort, conferring a significant survival benefit that is at least partly based on age. The widowed group, which showed the worst survival, also had the highest average age. We believe that age significantly confounds marital status and directly affects survival within these groups. This effect was likely confounded in Olarte et al., as the significant age differences seen between married, widowed, divorced, and domestic partners are lost in the original study when all were joined into a single category. Our stratified analysis provides a more nuanced understanding of how age interacts with the marital status effect seen in the SEER dataset.

Beyond age, the mechanisms underlying these sex‐specific differences in marriage are likely multifactorial. We demonstrated that marriage was more protective for men than women. Previous studies have suggested that men derive greater survival benefits from marriage in part due to the social support network provided by a spouse, as men are less likely than women to maintain broad support systems outside marriage [17, 18]. Married men (vs unmarried) are also more likely to seek timely medical evaluation and adhere to medical treatment due in part to greater social encouragement within the union [19, 20]. In contrast, for women, marriage may not confer an equivalent protective benefit as caregiver responsibilities are more often assumed by women within the household, attributing increased psychological and physical stress on women in relationships, offsetting the benefits of marital support [21, 22]. These patterns are consistent with broader findings showing the protective effects of marriage on health and survival are stronger for men than women [17, 23, 24, 25]. In head and neck cancer specifically, it has also been found that unmarried men had significantly higher hazards of death and were more likely to present with advanced disease than females [26]. These findings agree with our own findings in the SEER salivary gland dataset and suggest the potential mechanism for the observed differences. Our findings underscore the need to consider gendered social, behavioral, and support dynamics when interpreting the effects of marital status on outcomes.

This study also significantly improves upon Olarte et al. by using granular racial categories—including Asian/Pacific Islander and American Indian/Alaska Native patients—thus capturing disparities that would have been missed under the broader “Other” label used previously. Importantly, our results show better survival for Asian/Pacific Islander individuals, while there was no significant difference in survival between White and Black patients. Historically, there has been a growing divide in survival based on race, with White patients showing improvements in survival over time compared to Black patients in several different malignancies. Black patients have shown higher incidences of colorectal, breast, lung, and prostate malignancies with worse survival. This has been attributed to differences in delays in care, SES, insurance access, greater comorbidities, tumor characteristics, and treatment received [27, 28, 29, 30, 31]. A SEER analysis of MSGTs from 1988 to 2010 found that Hispanic patients had improved disease‐specific survival across all histopathological subtypes when compared to White and Black patients, while there was no difference between White and Black patients [32]. Another SEER study on cases from 1973 to 2009 found an overall and disease‐specific survival disadvantage for Black patients compared to White patients. However, multivariate adjusted Cox PH analysis indicated that this difference was not significant [9]. Two other studies, including a registry of 1573 patients in Florida from 1998 to 2002 [33] and a registry of 231 patients from 1990 to 2015 in Detroit [34], found no evidence for an impact of race on survival in univariate and multivariate analyses. A SEER study from 2000 to 2018 on MEC patients found that Asian or Pacific Islander patients had improved overall survival (HR = 0.5) on multivariate analysis [2]. Many of these correlations with higher Asian/Pacific Islander survival than other groups might be due to the significantly higher proportion of high‐income patients that Asian/Pacific Islander patients have in this cohort (Table 2) compared with every other race. These studies support findings in our analysis that show better survival for Asian Pacific Islanders while showing no statistically significant difference between White and Black patients.

Our study underscores the important and unequal role that SES, as measured by county‐level income, plays in survival for patients with MSGTs. Unlike Olarte et al., who categorized income into only two broad groups (above and below median) and found no significant multivariable association, we divided income into three groups to allow for more granularity in our analysis and compare the upper and lower limits of income to the group surrounding the median. We found that only individuals in the upper income group experienced a statistically significant survival benefit, with a 15% decrease in all‐cause mortality. Prior studies have demonstrated that SES is an independent predictor of survival in head and neck cancers [35]. The association of SES and survival has similarly been demonstrated in countries with universal healthcare, like Canada and Israel [36, 37], where they utilized census data to determine SES status. Iwata et al. found that areas with lower proportions of houses occupied and lower high school education rates were associated with worse 5‐ and 10‐year survival rates [34]. On multivariate analysis, residing in an area with worse educational attainment, but not the proportion of unoccupied houses, was a negative prognostic factor [34]. However, this study and others have found that there is no evidence of an impact of median household income or poverty levels on survival in salivary tumors [33, 34]. However, none of these studies further stratified income between sexes to investigate its impact on survival.

Importantly, the effect of SES was not uniform across sexes: while higher income conferred a survival benefit in male patients, this association was not observed in female patients. This divergence suggests that income may interact with other social determinants—such as employment patterns, health‐seeking behavior, or access to care—differently for men and women. In our results, male patients benefited more from higher income and marriage, whereas these factors were not protective for females.

We also suspect that societal pressure on men to be the breadwinners of the house may play a part in differences based on income. That role is fulfilled at higher household incomes, reducing stress and improving mortality for men alone. These findings support the growing body of literature indicating that income inequality remains a potent and sex‐dependent predictor of cancer outcomes, reinforcing the need for nuanced public health and clinical interventions that consider not just income, but also how sex modifies the impact of socioeconomic disadvantage. This may also explain why Asian/Pacific Islanders have increased survival both in our analysis and other studies [33].

Our study is one of the largest to date, looking at salivary gland malignancies composed of multi‐institutional, cross‐national data that support previous findings showing no significant difference between Black and White outcomes in demonstrated salivary gland malignancies. When stratifying on sex, the differences between Blacks and Whites continued to not be statistically significant in males, but showed a small difference in females. For males, racial differences may be erased due to tendencies to present with more advanced disease. On the other hand, racial differences in females may exist due to differences in access to preventative care, having a more significant impact on a younger age population with earlier disease status, as we discussed above.

### 4.1 Strengths, Limitations, and Future Direction

The SEER database has been shown to be a source of high‐quality cancer data nationwide. The large, standardized database allows studies like ours to limit bias by studying larger, more representative populations in the United States.

While this study substantially builds on insights into salivary gland malignancy, there are several limitations to our findings. First, we utilized a retrospective database built on registry data that contains its own limitations. Informational bias is possible as the SEER database is not complete with the full patient's medical history, social history, family history, specific therapies/regimens used, or past exposures. Clinically, cancer treatment and patient survival are also heavily impacted by staging criteria, which were largely not available in this dataset (47% missing values). Although this still yielded a large cohort with appropriate staging data available, it failed to meet statistical assumptions, and further research is needed to determine if the trends we see are affected by staging. Of note, the pathological diagnosis and classification of salivary gland tumors have evolved over this study period (2000–2021), particularly with the incorporation of immunohistochemical and molecular diagnostic techniques. These advances may have also influenced the accuracy or granularity of tumor classification over time, potentially affecting histologic subtype distribution and outcome interpretation. However, because SEER registry data are based on contemporaneous diagnostic criteria, each case reflects the standard practice at the time of diagnosis, and our analyses are consistent with those conventions. Further studies are needed to determine whether the population‐level trends observed over the last two decades are influenced by evolving staging guidelines and diagnostic refinements. Second, while the database tracks survival over time, it does not analyze quality of life outcomes or differences in surgical techniques over time. Future research is needed to determine these treatment‐specific outcomes and methods that may significantly affect quality of life in these populations. Third, data provided in the database is not always provided on a continuous basis. For example, income data in SEER are provided as county‐level estimates categorized into predefined bins, rather than as patient‐specific income values. As such, these aggregated measures may not accurately reflect individual SES, introducing potential for an ecological fallacy—where associations observed at the group level can mask individual confounders and variability, resulting in misleading inferences. Despite this, county‐level measures provide a useful proxy for socioeconomic context in population‐level analyses, allowing for a broad assessment of demographic and regional trends.

Further research into sex disparities is needed, given our findings and those in the literature. Grade‐specific determination of sex differences in the SEER database is needed to determine the underlying survival differences seen in men versus women. More data are necessary to determine the impact of race and social factors on major salivary gland malignancy outcomes at large [34]. Additional exploration is needed to evaluate the potential correlation between various health determinants and the major salivary gland malignancy population to improve care for these patients.

## 5 Conclusion

This study, using comprehensive SEER database analysis from 2000 to 2021, highlights the significant impact of demographic and socioeconomic factors on survival outcomes in patients with MSGTs. Key findings indicate that older age and male sex are associated with worse survival, while Asian/Pacific Islander race, female sex, and higher income may confer survival advantages. The rising incidence of salivary malignancy underscores the importance of continued research to address disparities in care that can lead to improved patient survival.

## Author Contributions

**Andrew R. Cunningham**: conceptualization, data curation, investigation, methodology, project administration, supervision, visualization, writing – original draft preparation, and writing – reviewing and editing; **Obaid Khurram**: investigation, conceptualization, software, visualization, formal data analysis, writing – reviewing and editing; **Hailey Lewis**: investigation, writing – original draft preparation, writing – reviewing and editing; **Nathan Barefoot**: investigation, data curation, writing – original draft preparation, writing – reviewing and editing; **Andrew W. Ju**: writing – reviewing and editing; **Sean Holmes**: writing – reviewing and editing; **Matthew S. Peach**: writing – reviewing and editing.

## Funding

The authors have nothing to report.

## Disclosure

The authors have nothing to report.

## Conflicts of Interest

The authors declare no conflicts of interest.

## Supporting information


**Figure S1:** Probability of survival (A) is shown across the entire cohort described. Survival based on primary tumor site across the major salivary glands is shown in (B), with parotid tumors having superior survival. The three main histologies (C) showed little difference in survival until 72 months, when MEC diverges. Grade (D) demonstrates higher grade disease has worse survival. Solid lines demonstrate the KM estimates with shaded areas representing 95% confidence intervals.

**Figure S2:** Illustrates the pairwise correlations among all independent variables utilized in the multivariable analyses derived from the SEER database. Variables include sex, race, marital status at diagnosis, income, and tumor grade level. The color gradient represents the strength and direction of correlations, ranging from **red (positive correlation)** to **blue (negative correlation)**, with **neutral tones indicating weak or no correlation**. Values along the diagonal denote perfect correlations (*r* = 1.0) for identical variables. Overall, minimal high‐correlation clustering was observed, but there were some correlations, including between males and higher grade, Asian and higher income.

**Table S1:** Numbers at risk across all Kaplan–Meier plots are demonstrated in Figure 3 and Figure S1.

**Table S2:** Numerical age illustrated across marital status and sex. Abbreviations include: std = standard deviation, sem = standard error of the mean.

## Data Availability

The data that support the findings of this study are available in the SEER database at https://seer.cancer.gov/data/access.html. These data were derived from the following resources available in the public domain: SEER Research Data, https://seer.cancer.gov/data/access.html.
