# Supplementary figures and images for: Sex and Health Disparities Impacts on Survival Rates for Patients With Major Salivary Gland Tumors
Source: Cancer Med. 2026 Feb 5;15(2):e71510. doi: 10.1002/cam4.71510 (PMC12877316; doi:10.1002/cam4.71510)

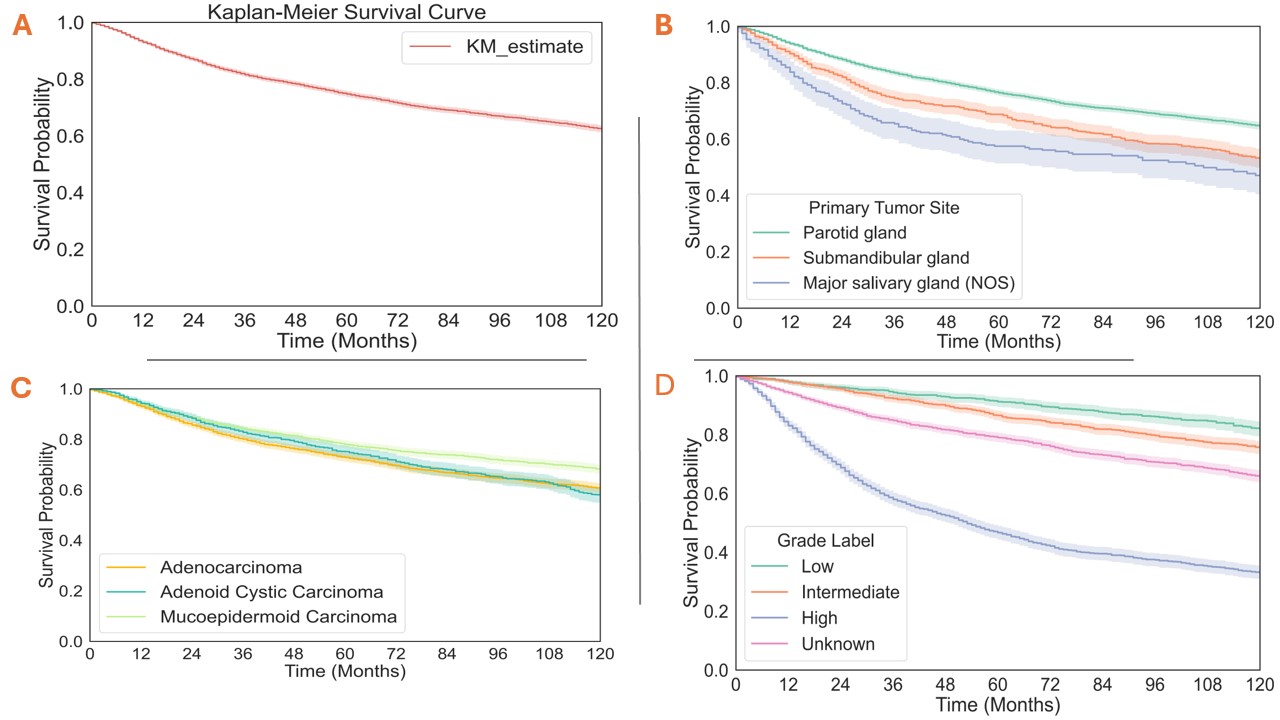

Supplement: Supplementary file 1 — Figure S1: Probability of survival (A) is shown across the entire cohort described. Survival based on primary tumor site across the major salivary glands is shown in (B), with parotid tumors having superior survival. The three main histologies (C) showed little difference in survival until 72 months, when MEC diverges. Grade (D) demonstrates higher grade disease has worse survival. Solid lines demonstrate the KM estimates with shaded areas representing 95% confidence intervals. [file CAM4-15-e71510-s001.jpg]

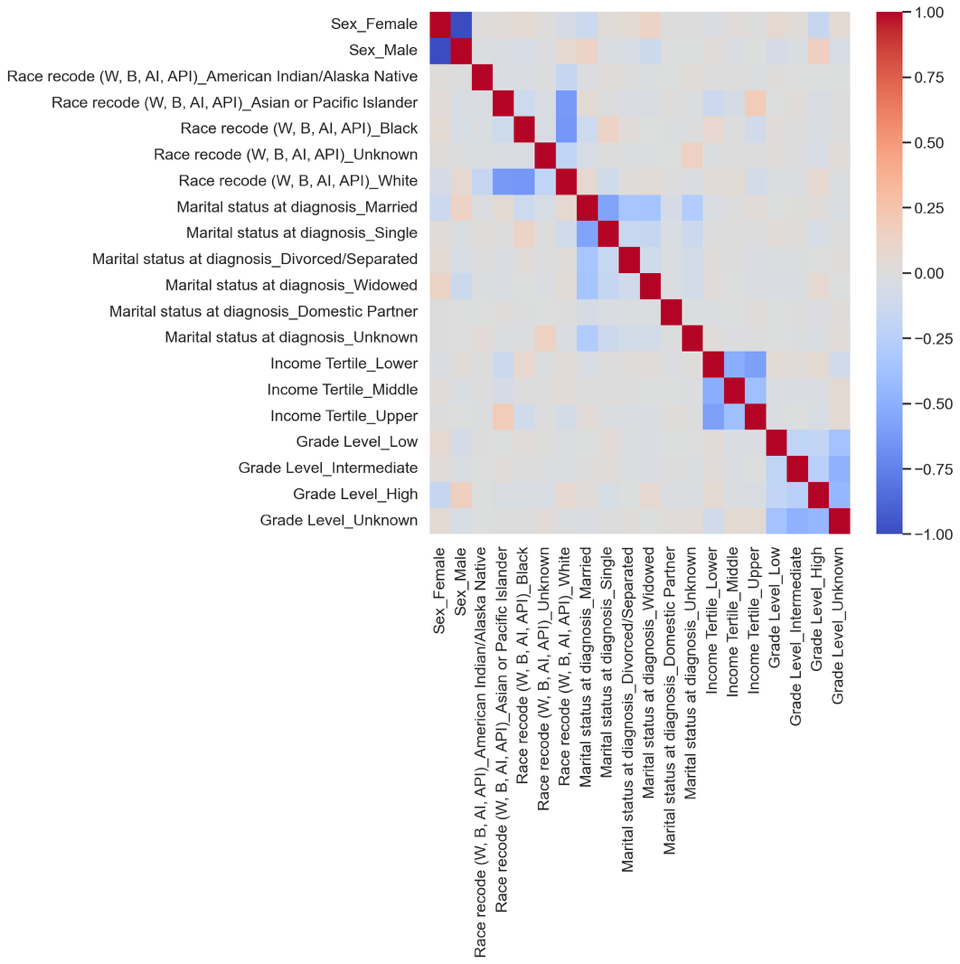

Supplement: Supplementary file 2 — Figure S2: Illustrates the pairwise correlations among all independent variables utilized in the multivariable analyses derived from the SEER database. Variables include sex, race, marital status at diagnosis, income, and tumor grade level. The color gradient represents the strength and direction of correlations, ranging from red (positive correlation) to blue (negative correlation), with neutral tones indicating weak or no correlation. Values along the diagonal denote perfect correlations (r = 1.0) for identical variables. Overall, minimal high‐correlation clustering was observed, but there were some correlations, including between males and higher grade, Asian and higher income. [file CAM4-15-e71510-s002.png]
