# Supplementary material for: Sex and Health Disparities Impacts on Survival Rates for Patients With Major Salivary Gland Tumors
Source: Cancer Med. 2026 Feb 5;15(2):e71510. doi: 10.1002/cam4.71510 (PMC12877316; doi:10.1002/cam4.71510)
Supplement: Supplementary file 4 — Table S2: Numerical age illustrated across marital status and sex. Abbreviations include: std = standard deviation, sem = standard error of the mean. [file CAM4-15-e71510-s003.docx]

**Supplemental 1 Title**: Table of Ages Stratified by Gender and Marital Status

|  |  | **Numerical Age** | | | |
| --- | --- | --- | --- | --- | --- |
|  |  | **mean** | **std** | **sem** | **median** |
| **Marital status at diagnosis** | **Sex** |  |  |  |  |
| **Married** | **Female** | 56.17555 | 15.20355 | 0.254598 | 57 |
|  | **Male** | 62.3225 | 14.84794 | 0.230429 | 64 |
| **Single** | **Female** | 42.6567 | 20.69157 | 0.527613 | 42 |
|  | **Male** | 46.22222 | 19.94543 | 0.561899 | 48 |
| **Divorced/Separated** | **Female** | 60.55967 | 13.12428 | 0.486085 | 61 |
|  | **Male** | 60.62448 | 12.89063 | 0.587152 | 60.5 |
| **Widowed** | **Female** | 76.39911 | 10.29059 | 0.343593 | 78 |
|  | **Male** | 77.60335 | 10.01423 | 0.529269 | 80 |
| **Domestic Partner** | **Female** | 47.95 | 12.56342 | 2.809266 | 46.5 |
|  | **Male** | 51.125 | 16.74465 | 4.186163 | 54.5 |
| **Unknown** | **Female** | 58.68 | 16.94955 | 0.799009 | 59.5 |
|  | **Male** | 59.88778 | 16.90754 | 0.844322 | 62 |
