# Supplementary material for: Sex and Health Disparities Impacts on Survival Rates for Patients With Major Salivary Gland Tumors
Source: Cancer Med. 2026 Feb 5;15(2):e71510. doi: 10.1002/cam4.71510 (PMC12877316; doi:10.1002/cam4.71510)
Supplement: Supplementary file 3 [file CAM4-15-e71510-s004.docx]

| Time (Months) | 0 | 12 | 24 | 36 | 48 | 60 | 72 | 84 | 96 | 108 | 120 |
|---|---|---|---|---|---|---|---|---|---|---|---|
| ***Sex*** | | | | | | | | | | | |
| **Female** | 7196 | 6313 | 5673 | 5084 | 4579 | 4124 | 3715 | 3317 | 2964 | 2666 | 2324 |
| **Male** | 6665 | 5623 | 4840 | 4219 | 3737 | 3314 | 2939 | 2612 | 2332 | 2063 | 1837 |
| ***Race*** | | | | | | | | | | | |
| **Asian or Pacific Islander** | 1401 | 1228 | 1099 | 975 | 878 | 782 | 699 | 624 | 562 | 499 | 421 |
| **Black** | 1479 | 1280 | 1121 | 978 | 876 | 781 | 689 | 616 | 539 | 482 | 427 |
| **White** | 10728 | 9215 | 8104 | 7180 | 6411 | 5736 | 5138 | 4575 | 4094 | 3661 | 3243 |
| ***Histology*** | | | | | | | | | | | |
| **Adenocarcinoma** | 5122 | 4373 | 3843 | 3384 | 3016 | 2717 | 2444 | 2207 | 1951 | 1729 | 1549 |
| **Adenoid Cystic Carcinoma** | 2252 | 1995 | 1746 | 1538 | 1373 | 1220 | 1094 | 963 | 862 | 769 | 664 |
| **Mucoepidermoid Carcinoma** | 5103 | 4449 | 3979 | 3567 | 3223 | 2893 | 2600 | 2332 | 2099 | 1885 | 1649 |
| ***Grade*** | | | | | | | | | | | |
| **Low** | 1858 | 1812 | 1763 | 1723 | 1680 | 1519 | 1356 | 1190 | 1060 | 921 | 791 |
| **Intermediate** | 2920 | 2814 | 2729 | 2626 | 2539 | 2250 | 2017 | 1801 | 1612 | 1445 | 1258 |
| **High** | 2528 | 2050 | 1672 | 1409 | 1254 | 1055 | 888 | 775 | 667 | 584 | 515 |
| **Unknown** | 6555 | 5260 | 4349 | 3545 | 2843 | 2614 | 2393 | 2163 | 1957 | 1779 | 1597 |
| ***Marital Status*** | | | | | | | | | | | |
| **Single** | 2797 | 2444 | 2185 | 1967 | 1774 | 1596 | 1448 | 1313 | 1189 | 1055 | 924 |
| **Married** | 7715 | 6727 | 5955 | 5271 | 4751 | 4290 | 3850 | 3443 | 3096 | 2790 | 2484 |
| **Divorced/Separated** | 1210 | 1041 | 910 | 806 | 705 | 614 | 549 | 478 | 416 | 373 | 317 |
| **Widowed** | 1252 | 969 | 795 | 675 | 577 | 497 | 419 | 359 | 307 | 260 | 219 |
| ***Primary Site*** | | | | | | | | | | | |
| **Major salivary gland, NOS** | 793 | 599 | 489 | 412 | 363 | 319 | 281 | 250 | 217 | 181 | 154 |
| **Parotid gland** | 10957 | 9545 | 8453 | 7517 | 6732 | 6030 | 5397 | 4812 | 4312 | 3860 | 3412 |
| **Submandibular gland** | 1891 | 1600 | 1395 | 1219 | 1086 | 972 | 868 | 778 | 686 | 623 | 533 |
| ***Income Group*** | | | | | | | | | | | |
| **Lower** | 5996 | 5223 | 4679 | 4212 | 3822 | 3462 | 3060 | 2702 | 2378 | 2098 | 1805 |
| **Middle** | 3510 | 2971 | 2565 | 2219 | 1943 | 1677 | 1520 | 1367 | 1231 | 1113 | 1011 |
| **Upper** | 4355 | 3742 | 3269 | 2872 | 2551 | 2299 | 2074 | 1860 | 1687 | 1518 | 1345 |
